# Surgical Management of Candy Cane Syndrome After Roux-en-Y Gastric Bypass: A Case Report Highlighting Diagnostic and Therapeutic Considerations

**DOI:** 10.7759/cureus.91725

**Published:** 2025-09-06

**Authors:** John Salib, Mark Salib, Olivia Mesdjian, Ujwal Yogesh, David Hernandez, Frederick Tiesenga

**Affiliations:** 1 School of Medicine, St. George's University School of Medicine, St. George's, GRD; 2 School of Medicine, St. George’s University School of Medicine, St. George’s, GRD; 3 School of Medicine, Washington University of Health and Science, Belize City, BLZ; 4 General Surgery, West Suburban Medical Center, Chicago, USA

**Keywords:** bariatric surgery, candy cane syndrome, case report, complication, laparoscopic roux-en-y gastric bypass, post-op complications, redundant limb, roux-en-y limb, surgical case reports, surgical management

## Abstract

Candy cane syndrome (CCS) is a rare complication of Roux-en-Y gastric bypass, caused by a redundant blind afferent limb at the gastrojejunostomy anastomosis. It typically presents with persistent postprandial abdominal pain, nausea, and vomiting, significantly impacting quality of life. We report the case of a 39-year-old woman who developed chronic upper abdominal discomfort two years after Roux-en-Y gastric bypass. Symptoms persisted despite conservative management, and CCS was ultimately identified as the underlying cause. Surgical resection of the redundant limb was performed, resulting in marked symptomatic relief. This case highlights the critical role of clinical suspicion and timely surgical intervention in the recognition and management of CCS while underscoring its significant impact on function and quality of life in post-bariatric surgery patients.

## Introduction

Obesity has become a global health crisis, with prevalence rates continuing to rise and leading to significant morbidity and mortality. Projections indicate that by 2026, approximately 42.7% of the world’s adult population will be overweight or obese, up from 40.9% in 2021 [1]. If current trends persist, the proportion of adults carrying excess weight could exceed half of the global adult population within the next few decades [2]. This mounting health burden has driven a parallel increase in the use of bariatric surgery as a long-term treatment strategy. Among surgical options, Roux-en-Y gastric bypass (RYGB) has emerged as one of the most effective interventions for sustained weight loss and improvement of obesity-related comorbidities [3]. In the United States alone, more than 250,000 bariatric surgeries are performed annually, with RYGB remaining a substantial contributor despite the growing preference for sleeve gastrectomy [4].

However, as with any complex surgical procedure, postoperative complications may arise, some presenting with nonspecific gastrointestinal symptoms and posing a diagnostic challenge [5]. One such complication is candy cane syndrome (CCS), a rare but increasingly recognized postoperative sequela of RYGB. CCS develops due to the creation of an excessively long blind afferent limb at the gastrojejunostomy anastomosis, which can result in functional obstruction and symptoms such as abdominal pain, nausea, vomiting, and intolerance to solid foods. Reported prevalence ranges from 1% to 5% among post-bariatric surgery patients, though true incidence is likely underestimated due to misattribution of symptoms to more common postoperative conditions [6,7]. Despite its rarity, CCS can markedly diminish quality of life, and diagnostic delays often contribute to prolonged morbidity.

Given the continued rise in bariatric surgery worldwide, incidences of CCS are expected to increase. Therefore, heightened clinical awareness is crucial for timely recognition and effective intervention. We present the case of a 39-year-old woman who developed persistent left upper quadrant and epigastric pain, nausea, and vomiting following RYGB. After a thorough diagnostic evaluation, she was diagnosed with CCS and underwent surgical revision, resulting in significant symptomatic and functional improvement.

## Case presentation

The patient is a 39-year-old woman with a history of RYGB performed in 2022, presenting to the surgical outpatient clinic with a two-year history of upper abdominal pain. The pain was postprandial in nature and associated with nausea and intermittent vomiting. She reported that her symptoms persisted despite multiple interventions, including antacids, digestive enzymes, antiemetics, proton pump inhibitors, antispasmodics, and lifestyle modifications. She denied cardiopulmonary or systemic symptoms, including shortness of breath, cough, chest pain, palpitations, fever, or chills.

Her past medical history was significant for hypothyroidism, sleep apnea, asthma, and deep vein thrombosis. She reported allergies to sulfonamides, phenazopyridine, and several antidepressants. Her surgical history included tubal ligation, uterine fibroid ablation, tonsillectomy, sleeve gastrectomy, laparoscopic gastric band placement and removal, RYGB, umbilical hernia repair, and laparoscopic cholecystectomy.

The patient appeared well nourished and cooperative but was notably fatigued and in mild distress due to abdominal discomfort. Vital signs were within normal limits. On abdominal examination, the abdomen was soft and nondistended, with multiple well-healed surgical scars reflecting her prior extensive abdominal procedures. Palpation revealed moderate tenderness localized to the left upper quadrant and epigastric region, both superficially and with deep pressure. There was no rebound tenderness, guarding, or rigidity, and the Murphy sign was negative. Bowel sounds were normoactive throughout all quadrants, and no palpable masses, hepatosplenomegaly, or fluid wave were detected. Examination of the cardiopulmonary system was unremarkable, with normal heart sounds and clear lung fields. Extremities were without edema, cyanosis, or signs of deep vein thrombosis.

Laboratory evaluation (Table 1), including a comprehensive metabolic panel, liver function tests, and complete blood count with differential, revealed values within normal limits. Electrolytes, renal function, liver enzymes, and hematologic indices were all unremarkable, suggesting the absence of systemic inflammation, infection, or significant metabolic disturbance. These findings are consistent with typical presentations of CCS, in which the underlying pathology is mechanical rather than biochemical. While chronic nausea or vomiting can occasionally lead to electrolyte imbalances or mild nutritional deficiencies, the patient’s laboratory results indicated adequate nutritional status and metabolic stability, supporting the conclusion that her symptoms were unlikely to be secondary to systemic illness.

**Table 1:** Laboratory Results on Admission. Laboratory evaluation on admission, including a comprehensive metabolic panel, liver function tests, and complete blood count with differential. All values were within normal limits, consistent with the mechanical nature of candy cane syndrome and absence of systemic metabolic or inflammatory disturbances.

Category	Test	Result	Reference Range
Comprehensive metabolic panel (CMP)	Sodium (Na)	138 mmol/L	135–145 mmol/L
	Potassium (K)	3.8 mmol/L	3.5–5.0 mmol/L
	Chloride (Cl)	102 mmol/L	98–107 mmol/L
	Carbon dioxide (CO₂)	25 mmol/L	22–29 mmol/L
	Blood urea nitrogen (BUN)	14 mg/dL	7–20 mg/dL
	Creatinine (Cr)	0.9 mg/dL	0.6–1.3 mg/dL
	BUN/creatinine ratio	16	10–20
	Glucose	96 mg/dL	70–110 mg/dL
	Calcium (Ca)	9.4 mg/dL	8.5–10.5 mg/dL
	Total protein	7.2 g/dL	6.0–8.3 g/dL
	Albumin (Alb)	4.1 g/dL	3.5–5.0 g/dL
Liver function test (LFT)	Aspartate aminotransferase (AST)	28 U/L	10–40 U/L
	Alanine aminotransferase (ALT)	32 U/L	7–56 U/L
	Alkaline phosphatase (ALP)	85 U/L	44–147 U/L
	Total bilirubin (Tbili)	0.8 mg/dL	0.1–1.2 mg/dL
Complete blood count (CBC)	White blood cell (WBC)	6.2 ×10³/µL	4.0–10.5 ×10³/µL
	Red blood cell (RBC)	4.5 ×10⁶/µL	4.2–5.4 ×10⁶/µL
	Hemoglobin (Hb)	13.5 g/dL	12.0–16.0 g/dL
	Hematocrit (Hct)	40 %	36–46 %
	Mean corpuscular volume (MCV)	89 fL	80–100 fL
	Mean corpuscular hemoglobin (MCH)	30 pg	27–33 pg
	Mean corpuscular hemoglobin concentration (MCHC)	34 g/dL	32–36 g/dL
	Red cell distribution width (RDW)	13 %	11.5–14.5 %
	Platelets (Plt)	220 ×10³/µL	150–450 ×10³/µL
	Mean platelet volume (MPV)	9.2 fL	7.5–11.5 fL
Complete blood count differential (CBC Diff)	Neutrophils (%)	58 %	40–70 %
	Lymphocytes (%)	32 %	20–40 %
	Monocytes (%)	6 %	2–8 %
	Eosinophils (%)	3 %	1–4 %
	Basophils (%)	1 %	0–1 %
	Neutrophils absolute	3.6 ×10³/µL	1.8–7.5 ×10³/µL
	Lymphocytes absolute	2.0 ×10³/µL	1.0–4.0 ×10³/µL
	Monocytes absolute	0.35 ×10³/µL	0.2–0.8 ×10³/µL
	Eosinophils absolute	0.15 ×10³/µL	0.0–0.5 ×10³/µL
	Basophils absolute	0.05 ×10³/µL	0.0–0.1 ×10³/µL

Over the preceding two years, pharmacologic therapies and lifestyle modifications failed to provide symptomatic relief for the patient’s abdominal discomfort. She underwent a colonoscopy and a computed tomography (CT) scan of the abdomen and pelvis with and without contrast, both of which were inconclusive. A small bowel follow-through (SBFT) was also performed and revealed no abnormalities. Subsequently, an esophagogastroduodenoscopy (EGD) demonstrated a long, redundant blind limb at the gastrojejunostomy anastomosis measuring approximately 6-8 cm. The blind limb contained retained food material and exhibited erythema, edema, and mucosal inflammation, suggesting chronic irritation and stasis. The gastric pouch and efferent jejunal limb showed no evidence of ulceration or obstruction. These findings supported the diagnosis of CCS and provided a clear rationale for surgical resection of the blind limb to relieve mechanical stasis and reduce mucosal inflammation.

**Figure 1:**
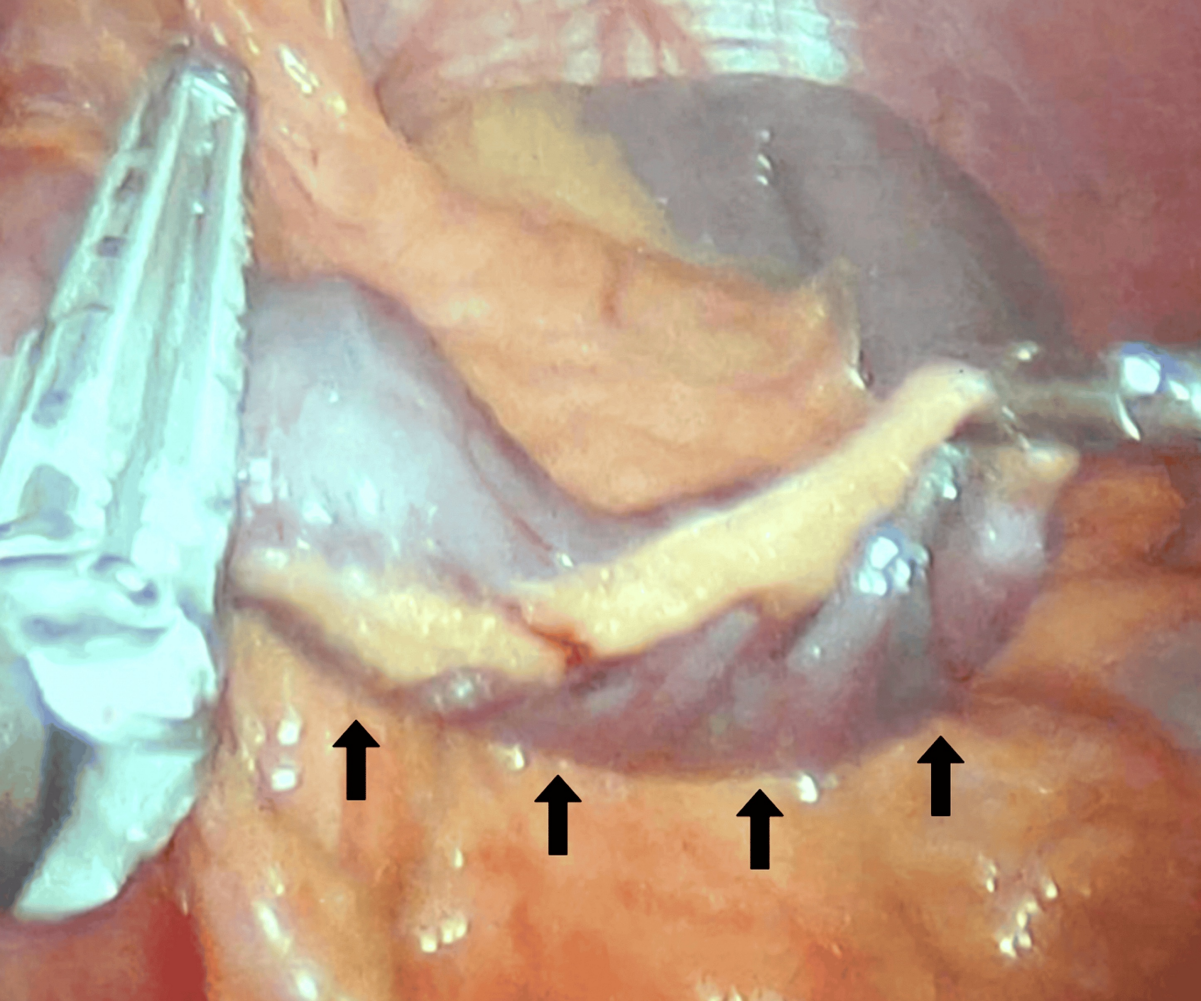
Laparoscopic View of a Redundant Afferent Limb in Candy Cane Syndrome. Intraoperative laparoscopic image showing the redundant blind afferent limb (indicated by arrows) adjacent to the gastrojejunostomy. This elongated segment is characteristic of candy cane syndrome and can contribute to postprandial pain, nausea, and reflux symptoms.

Given the diagnostic uncertainty and the patient’s persistent postprandial abdominal discomfort that had not responded to pharmacologic or lifestyle interventions, a diagnostic laparoscopy was undertaken to allow comprehensive evaluation of the gastrojejunal anatomy. Intraoperatively, the surgical team identified a redundant “candy cane” Roux limb (Figure 1) at the gastrojejunostomy anastomosis, which measured approximately 6-8 cm, consistent with preoperative endoscopic findings. The limb was associated with dense adhesions to surrounding tissues, which contributed to localized stasis and mechanical obstruction.

**Figure 2:**
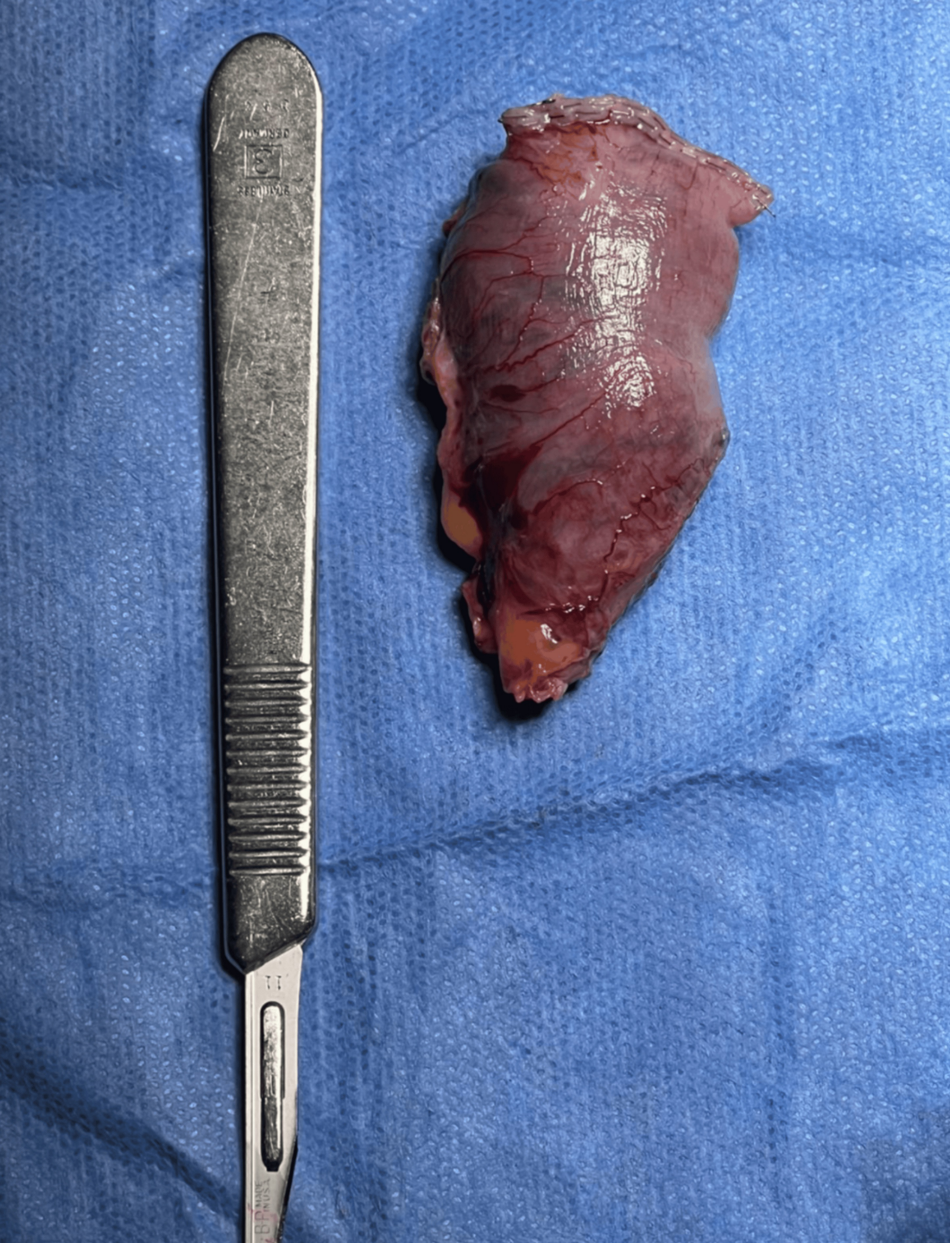
Resected Blind Afferent Limb in Candy Cane Syndrome. Gross specimen of a 6–8 cm redundant blind afferent limb excised during revision surgery for candy cane syndrome. The elongated segment of jejunum, adjacent to the gastrojejunostomy, can act as a functional reservoir, leading to postprandial discomfort, nausea, and reflux symptoms. A scalpel is placed adjacent for size reference.

After careful dissection, adhesiolysis was performed to free the redundant segment, and the blind limb was resected (Figure 2) to eliminate the reservoir for food and digestive secretions. This intervention aimed to provide both symptomatic relief, by reducing postprandial pain, nausea, and vomiting, and functional restoration of regular gastrointestinal transit. The procedure was completed without intraoperative complications, and the remainder of the Roux-en-Y anatomy appeared intact and well perfused. Postoperative management focused on gradual diet advancement, monitoring for early complications, and optimization of nutritional status.

**Figure 3:**
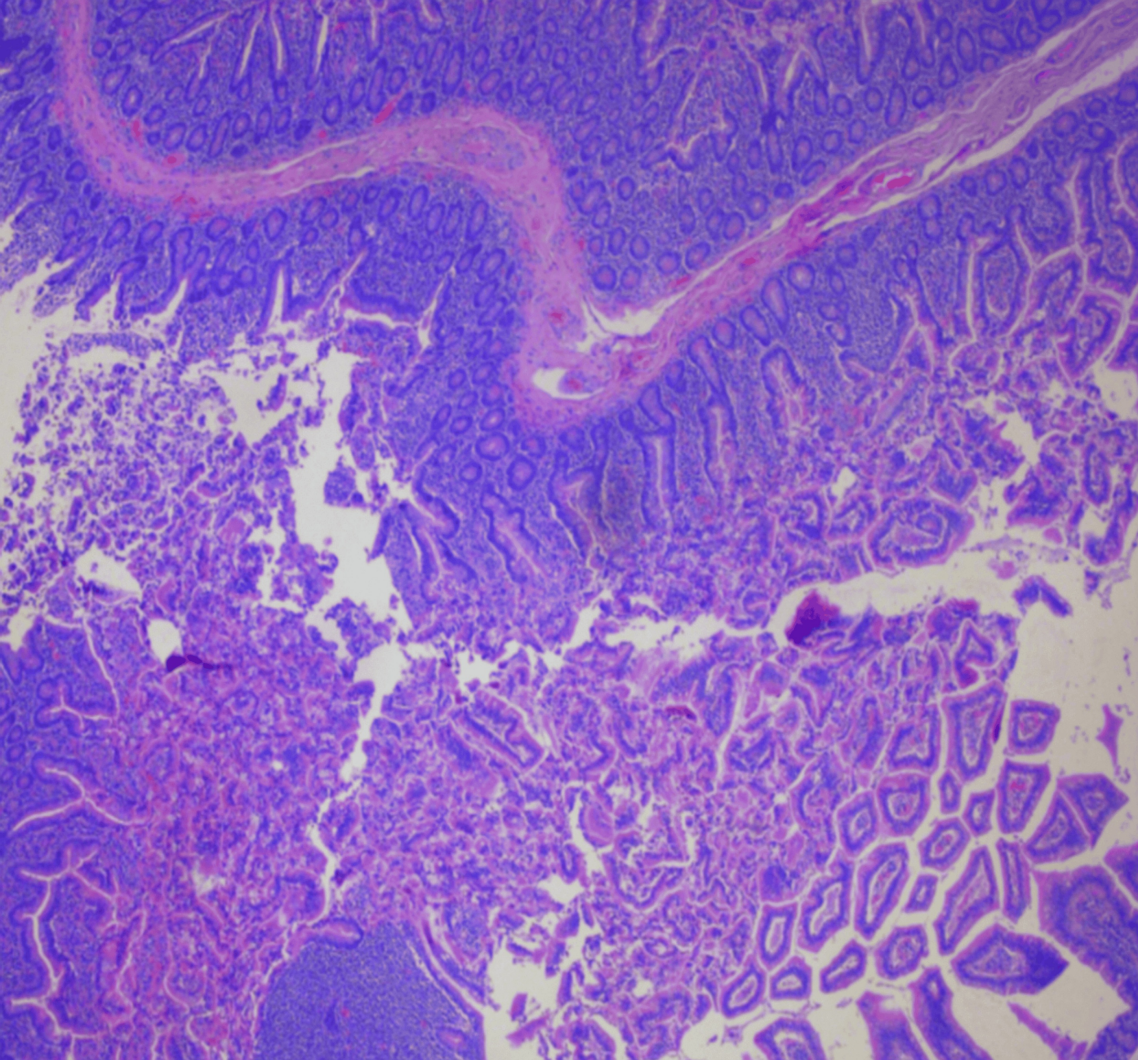
Hematoxylin and Eosin Stained Section of the Resected Redundant Candy Cane Roux Limb (×100 Magnification). The section demonstrates preserved villous architecture with glandular and crypt structures highlighted by hematoxylin and eosin staining. Increased contrast between nuclei and cytoplasm/stroma allows clear visualization of mucosal organization.

Histopathologic examination of the resected gastrojejunal tissue demonstrated focal chronic inflammation with reactive epithelial changes. The serosal surface appeared tan-pink and pale, while the mucosa was tan, viable, and unremarkable. Immunohistochemical staining was negative for Helicobacter pylori, and there was no evidence of intestinal metaplasia, dysplasia, or malignancy (Figure 3). These findings are consistent with chronic irritation related to stasis in the redundant blind limb rather than a primary infectious or neoplastic process, corroborating the clinical diagnosis of CCS.

Following surgery, the patient was closely monitored and stabilized, demonstrating adequate pain control, hemodynamic stability, and bowel function. She was gradually advanced to an oral diet, which she tolerated without complications, and was subsequently discharged in stable condition. At follow-up in the outpatient setting a few weeks later, the patient reported substantial improvement in her postprandial abdominal symptoms, including a marked reduction in pain, nausea, and vomiting, consistent with successful resection of the redundant Roux limb.

## Discussion

Although cases of CCS are becoming increasingly recognized in the context of the rising number of RYGB procedures, it remains a rare and underdiagnosed complication [7]. The true prevalence is difficult to establish, as many patients may remain asymptomatic for extended periods or be misdiagnosed with alternative postoperative sequelae. Published literature suggests that CCS is likely underreported, with some estimates indicating a prevalence of approximately 3-5% among post-bariatric patients undergoing revisional surgery [8]. This underestimation is particularly concerning given the steadily increasing prevalence of obesity worldwide and the projected growth in bariatric procedures, which in turn increases the likelihood that clinicians will encounter this syndrome in practice. This case contributes to the growing body of evidence emphasizing the importance of early recognition of CCS to minimize diagnostic delays and prevent prolonged morbidity.

The pathophysiology of CCS is rooted in the formation of a redundant blind afferent limb at the gastrojejunostomy anastomosis. This redundant limb acts as a reservoir, leading to stasis of ingested food and secretions, mechanical distension, and functional obstruction [4]. Over time, this produces motility disturbances and elevated intraluminal pressures that translate into postprandial pain, nausea, vomiting, and bloating. In severe cases, persistent stasis may compromise nutritional status and negate the intended benefits of bariatric surgery as a result of inadequate oral intake [5,6]. In our patient, this pathophysiologic process explained her chronic left upper quadrant and epigastric pain, nausea, and vomiting, which had persisted for nearly two years following her gastric bypass. Importantly, her symptoms were not only debilitating but also eroded her quality of life and raised the risk of malnutrition, underscoring the functional burden CCS can impose when left untreated.

Diagnosis of CCS remains a considerable challenge due to its nonspecific presentation and overlap with other post-bariatric complications (Table 2), such as marginal ulceration, anastomotic stricture, or internal hernia. Conventional diagnostic modalities, including CT scans, barium swallow studies, and upper endoscopy, have been reported to have limited sensitivity, often ranging from only 30% to 60% [8]. This diagnostic uncertainty frequently leads to delays in care, with patients undergoing extensive evaluations before CCS is ultimately identified. Our patient followed this trajectory: despite repeated imaging and endoscopic workup, no alternative diagnosis could account for her symptoms, and CCS was confirmed only after persistent evaluation and clinical suspicion. This highlights the importance of considering CCS early in the differential diagnosis of post-Roux-en-Y patients with chronic abdominal complaints, even in the absence of conclusive imaging.

**Table 2:** Comparative Clinical Features of Candy Cane Syndrome and Common Differential Diagnoses. Comparison of CCS with other post-Roux-en-Y complications. While these conditions may share symptoms such as abdominal pain, nausea, and vomiting, key differences in presentation and diagnostic findings help distinguish CCS.

Condition	Typical Presentation	Similarity to CCS	Key Differences From CCS
Candy cane syndrome (CCS)	Postprandial epigastric or right upper quadrant pain, nausea, vomiting, food intolerance, occasional weight loss		Primary disorder of the blind afferent limb; symptoms improve after surgical resection of the redundant limb
Marginal ulcer	Epigastric pain, nausea, vomiting, possible gastrointestinal bleeding, anemia	Pain and nausea may mimic CCS	Endoscopy shows mucosal ulceration; bleeding and anemia are common, which are not features of CCS
Anastomotic stricture	Progressive intolerance to solids, vomiting undigested food, and weight loss	Food intolerance and vomiting resemble CCS	Narrowed lumen on endoscopy; balloon dilation relieves symptoms, unlike CCS
Internal hernia	Intermittent crampy abdominal pain, bloating, nausea, and possible acute obstruction	Episodic abdominal pain and nausea may resemble CCS	Pain is often acute and severe; CT may show mesenteric swirl; surgical repair resolves the hernia, not a redundant limb
Small bowel obstruction (adhesions/kinking)	Abdominal pain, distension, vomiting, and obstipation	Vomiting and abdominal discomfort similar to CCS	Imaging reveals dilated loops/air-fluid levels; mechanical obstruction is distinct from blind limb stasis
Gastrogastric fistula	Abdominal pain, reflux, weight regain, persistent ulcers	Chronic abdominal symptoms may overlap with CCS	Contrast studies/endoscopy reveal fistula, associated with weight regain, unlike CCS, which often causes weight loss

Management of CCS is dictated by symptom severity and the degree of functional impairment. Conservative measures such as dietary modification or medical therapy are rarely sufficient for lasting symptom control. Definitive treatment involves surgical resection of the blind afferent limb, which has demonstrated excellent outcomes, with symptom resolution rates approaching 90-95% in reported case series [9]. Emerging endoscopic therapies, such as creation of a jejunojejunostomy using lumen-apposing metal stents (LAMS), have shown promise as minimally invasive alternatives, but data remain limited and follow-up is short-term [10].

A 2017 case series reported that 94% of patients experienced complete resolution of symptoms following surgical resection of the blind afferent limb [9]. Between 2016 and 2018, a novel endoscopic approach using a LAMS to create a jejunojejunostomy was performed in four patients, achieving symptom resolution in all cases within six weeks [10]. While the LAMS approach offers a minimally invasive option with rapid short-term improvement, surgical resection remains the gold standard, providing durable symptom relief and definitive correction of the anatomical abnormality. The limited sample size and short follow-up in LAMS studies highlight the need for further research before widespread adoption. In contrast, surgical outcomes have been consistently reproducible, underscoring the continued role of operative management as the primary treatment modality for CCS.

In our patient, surgical resection was pursued after extensive evaluation confirmed the diagnosis. Postoperatively, she experienced marked relief of her symptoms, reinforcing existing evidence that surgical intervention remains the gold standard for both symptomatic and functional recovery.

## Conclusions

CCS remains a rare but clinically significant complication of RYGB, often presenting with nonspecific postprandial abdominal pain, nausea, and vomiting. This case highlights the diagnostic challenges inherent to CCS, including its overlap with other postoperative complications and the limited sensitivity of conventional imaging and endoscopic studies. The patient’s chronic symptoms, together with endoscopic and intraoperative evidence of a redundant blind afferent limb with mucosal inflammation, underscore the need for a high index of suspicion when evaluating post-bariatric patients with persistent upper abdominal discomfort. Surgical resection of the redundant limb provided both symptomatic relief and restoration of gastrointestinal function, reinforcing the role of operative management as the definitive treatment for CCS. Early recognition and intervention are crucial for preventing prolonged morbidity, improving patient quality of life, and optimizing the functional outcomes of bariatric surgery.
